# Comparison of the Intensity of Biofilm Production by Oral Microflora and Its Adhesion on the Surface of Zirconia Produced in Additive and Subtractive Technology: An In Vitro Study

**DOI:** 10.3390/ma17061231

**Published:** 2024-03-07

**Authors:** Wojciech Frąckiewicz, Agata Pruss, Marcin Królikowski, Paweł Szymlet, Ewa Sobolewska

**Affiliations:** 1 Department of Dental Prosthetics, Faculty of Medicine and Dentistry, Pomeranian Medical University in Szczecin, 70-111 Szczecin, Poland; 2 Department of Laboratory Medicine, Faculty of Medicine and Dentistry, Pomeranian Medical University in Szczecin, 70-111 Szczecin, Poland; 3 Department of Manufacturing Engineering, Faculty of Mechanical Engineering and Mechatronics, West Pomeranian University of Technology in Szczecin, 70-310 Szczecin, Poland

**Keywords:** three-dimensional, additive-manufacturing, zirconium, 3D printing, biofilm, microflora, saliva, bacteria, dentistry, prosthodontics

## Abstract

Background: This in vitro study set out to find out how well oral cavity-dwelling bacteria can form biofilms and adhere on the surfaces of zirconium oxide samples created by 3D printing and milling technologies. Methods: 5 strains of microorganisms were used for the study, and 40 zirconium oxide samples were prepared, which were divided into two groups (*n* = 20)—20 samples produced using removal technology comprised the control group, while 20 samples produced by 3D printing technology comprised the test group. The prepared samples were placed in culture media of bacteria and fungi that naturally occur in the oral cavity. Then, the intensity of biofilm build-up on the samples was determined using qualitative and quantitative methods. The results for both materials were compared with each other. Results: No variations in the degree of biofilm deposition on zirconium oxide samples were found for the microorganisms *Streptococcus mutans*, *Pseudomonas aeruginosa*, *Enterococcus faecalis*, and *Staphylococcus aureus*. For *Candida albicans* fungi, more intense biofilm deposition was observed on samples made using 3D printing technology, but these differences were not statistically significant. Conclusion: The biofilm accumulation intensity of ceramics produced by additive technology is comparable to that of milled zirconium oxide, which supports the material’s broader use in clinical practice from a microbiological perspective. This ceramic has demonstrated its ability to compete with zirconium oxide produced by milling techniques in in vitro experiments, but sadly, no in vivo tests have yet been found to determine how this material will function in a patient’s oral cavity.

## 1. Introduction

Prosthetics and conservative dentistry are fields of dentistry that deal with the reconstruction of a patient’s damaged teeth. Tooth reconstruction materials are often used as direct restorations, made by a dentist in a dental office, or indirect restorations, made in a dental laboratory [1]. Direct restorations are usually made of composite materials, glass ionomers, or their variants, such as ormocers or compomers [2], so they can be made in one visit. Performing indirect restorations such as onlays, overlays, inlays, endocrowns, or, in the case of prosthetics, crowns and bridges requires more work and time, but the durability is greater if such restorations are properly planned and made [3]. The materials used for such work in modern dentistry are usually various types of ceramics. The most widely used is zirconium dioxide, which not only allows for good aesthetic effects, but also has very high mechanical parameters [4,5]. Originally, due to the lack of variation in the shades of zirconium oxide, it was used only as bases for permanent restorations, in which its contact with the oral environment was limited to the cervical area and the part adjacent to the tooth. Currently, when manufacturers are able to provide zirconium oxide in the form of milling blocks, which can not only be in the appropriate color, but also their shade which can change along the entire length of the restoration; zirconium oxide can be used as the only material constituting a prosthetic restoration, without the need for covering ceramics with glaze [6]. Recently, zirconium has also been used in implant prosthetics as a material for making implants, which resulted in its contact not only with the tooth surface, but also with the patient’s bone tissues [7]. With the development and increasing use of zirconium oxide in dentistry, a problem emerged in the form of exposure of this material to conditions in the oral cavity, including the deposition of various types of microorganisms on its surface.

There are over 700 species of fungi and bacteria in the oral cavity [8]. Most of them are benign microorganisms that protect teeth and oral tissues while also helping in the digestion of meals. Surfaces in the oral cavity, such as cheeks, tongue, teeth, as well as dental restorations, are places where microflora elements accumulate, which, if not cleaned in an appropriate period of time, can lead to gum disease and tooth damage. Dental health is extremely threatened by carbohydrate-degrading bacteria, including *Streptococcus mutans* and *Streptococcus gordonii* [9]. For growth, they primarily use carbohydrates that remain in the mouth after eating if we do not clean it thoroughly. These bacteria turn carbohydrates into acids, which change the pH in the oral cavity, promoting the development of caries and enamel damage. In humans, fungi also occur in the oral cavity, constituting the physiological flora of the oral cavity and usually not causing symptoms even throughout life. However, some people may develop candidiasis if their immunity is weakened or if they are treated with antibiotics. The fungus most often causing oral mycosis is *Candida albicans*, but there are also infections caused by yeasts of the species *Candida krusei*, *Candida tropicalis*, and *Candida glabrat* [10].

With the development of new technologies, new concepts for the production of zirconium oxide were presented. Currently, the most popular technology is CAD/CAM (computer-aided design and computer-aided manufacturing), which involves milling of restoration from a block or disk, which is previously designed based on a scan of the oral cavity made in a dentist’s office [11]. In recent years, scientists have also become interested in the additive manufacturing of zirconium oxide using 3D printing. This printing is performed using various technologies, and the most popular ones include LCM (Lithography-based Ceramic Manufacturing), SLA (Stereolithography), and DLP (Digital Light Processing) [12,13]. However, these technologies are not widely used in clinical practice, despite their many advantages, such as printing in various porosities and densities [14], as well as the possibility of producing geometrically complex structures [15].

Dental materials used in the production of prosthetic restorations are exposed to conditions in the oral cavity, including the influence of bacteria and fungi. Materials that have been known for years in dental work, such as zirconium oxide, composite resins [16], or lithium disilicate [17], meet these requirements, and studies dealing with them often mention the relationship that the adhesion of bacteria to the surface increases with an increase in their roughness. This is caused, among other things, by more difficulties in cleaning and an increase in the surface of contact with the external environment. In the case of zirconium oxide produced using additive technology, we can find publications dealing with its mechanical parameters, such as compressive strength or hardness [18,19]. In turn, when it comes to the usefulness and exposure of this material to the conditions in the oral cavity, there are publications on zirconium oxide produced using the milling technique [20], but this issue is not discussed in relation to the material produced using the additive technique.

The aim of the study is to analyze the influence of oral microflora on the formation of biofilm and the adhesion of bacteria and fungi to the surface of milled and 3D printed zirconium oxide. Due to the small amount of research on this topic, the authors want to compare whether there are differences between the materials significant enough to ensure the superiority of one material over the other. At the beginning of the research, the null hypothesis was assumed that the given materials did not show any differences.

## 2. Materials and Methods

### 2.1. Zirconium Oxide Samples

**Figure 1:**
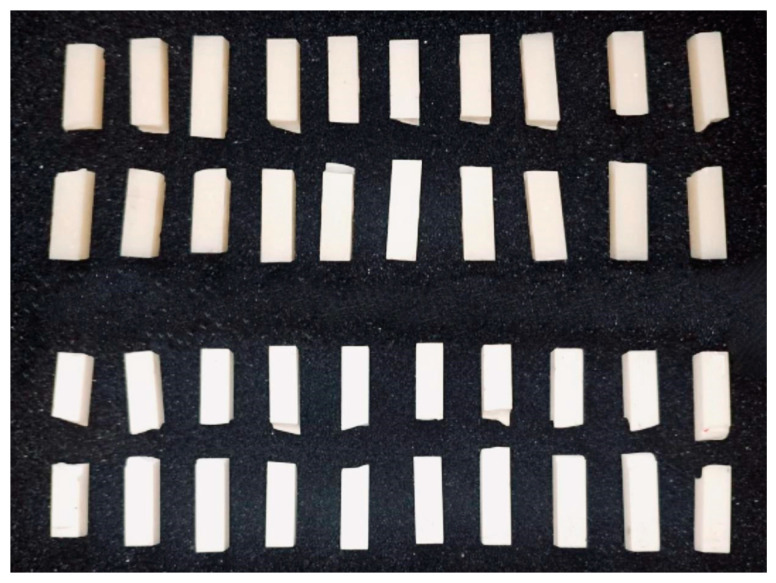
Samples prepared by milling—SM group (**top**)—and by 3D printing—AM group (**bottom**).

Before microbiological tests, surface measurements were performed on zirconium oxide samples to examine the roughness of its surface [21]. To make them, 20 bars with dimensions of 30 × 5 × 4 mm were used in two technologies, 10 for subtractive technology (SM group) and additive technology (AM group). Special software for creating dental designs was used to design them (Exocad Rijeka 3.1, Exocad GmbH, Darmstadt, Germany), and then STL files of samples for testing were exported. Mechanical tests were also performed, where, during bending strength testing, the bars cracked, which divided each of them into two parts, increasing the number of samples for a given technology by twofold (*n* = 20). The samples for microbiological tests (Figure 1) were not processed to see how their surface directly after the sintering process would behave in conditions similar to those in the oral cavity.

Monolithic disks of yttrium-stabilized zirconium oxide (IPS e.max ZirCAD LT, Ivoclar Digital, Schaan, Liechtenstein) in shade A2 (*n* = 20) were used to create the samples for the SM group. They were created using a 4-axis milling and grinding machine (Z4, VHF, Ammerbuch, Germany), with the device software (DentalCAM 7.08, VHF, Germany) being used to construct the sample shape originally. Following milling, the samples were sintered at a temperature of 1530 degrees Celsius in an oven (HT-S Speed, Mihm-Vogt, Stutensee, Germany), and then they were cooled gradually to room temperature in compliance with the manufacturer’s instructions. The bars were developed with a magnification of 1.218 since the sintering process reduces the size of zirconium oxide.

A 3D printer (Ceramaker C900, 3D Ceram Sinto, Côte, France) was used to create samples for the AM group (*n* = 20). The polymerization process is carried out by this printer using a 355 nm UV laser positioned at the base of the printer platform, with a sintering point diameter of around 35 µm. Stereolithography technology, which is predicated on the suspension photopolymerization procedure, is used for printing. The components were printed using a material (3DMix ZR3-F01, 3D Ceram Sinto, Côte, France) made of photosensitive polymer—that is, organic materials burned during the debinding process—and ceramic powder, which was medical-grade zirconium oxide. The thickness of the printed material was 25 μm per layer. Following the sintering process, the printed samples were progressively heated in an oven (HTL 20/17, Thermconcept GmbH, Bremen, Germany) to 1450 degrees Celsius. They were then cooled to room temperature in compliance with the manufacturer’s guidelines, leaving just the ceramic structure.

### 2.2. Bacterial and Fungal Strains

**Figure 2:**
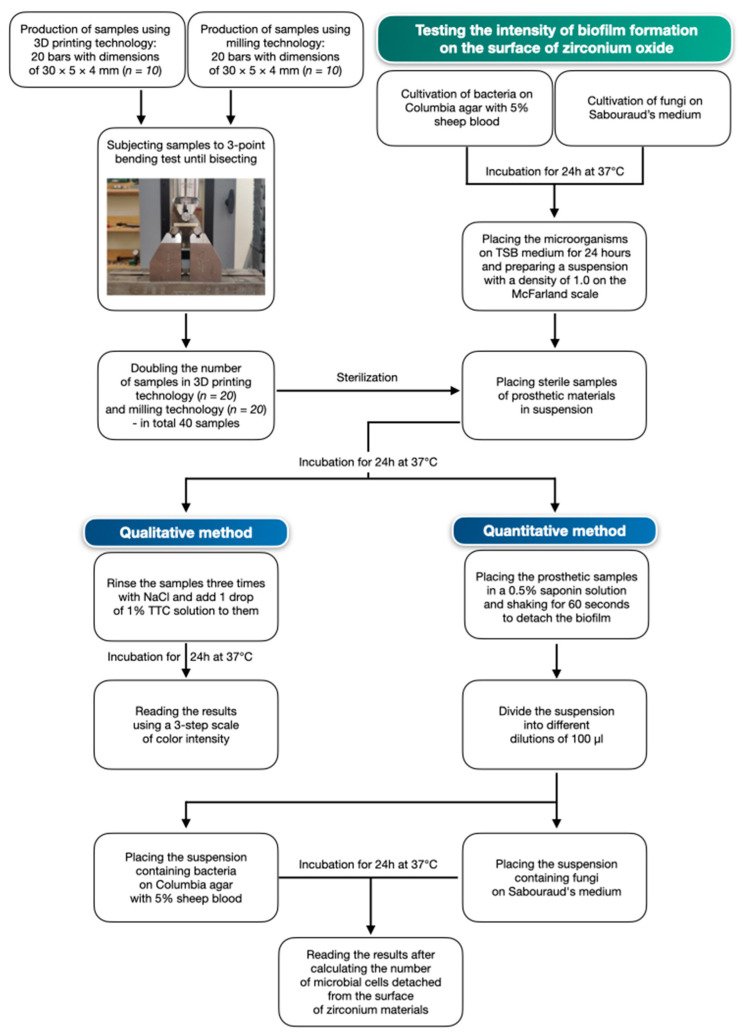
Research methodology.

The methodology for examining the intensity of biofilm formation is presented in Figure 2.

*Streptococcus mutans* ATCC 35668, *Pseudomonas aeruginosa* ATCC 27853, *Enterococcus faecalis* ATCC 29212, *Staphylococcus aureus* ATCC 25923, and *Candida albicans* ATCC 10231 (Argenta, Poznań, Poland) were the reference strains utilized in the study. Bacterial culture was conducted on Columbia agar medium with 5% sheep blood (bioMérieux, Craponne, France); yeast culture was conducted on Sabouraud medium (bioMérieux, Craponne, France). The incubation process lasted 24 h at 37 °C under aerobic conditions.

### 2.3. Study of Biofilm Formation on the Surface of Ceramic Materials

#### 2.3.1. Qualitative Method

**Figure 3:**
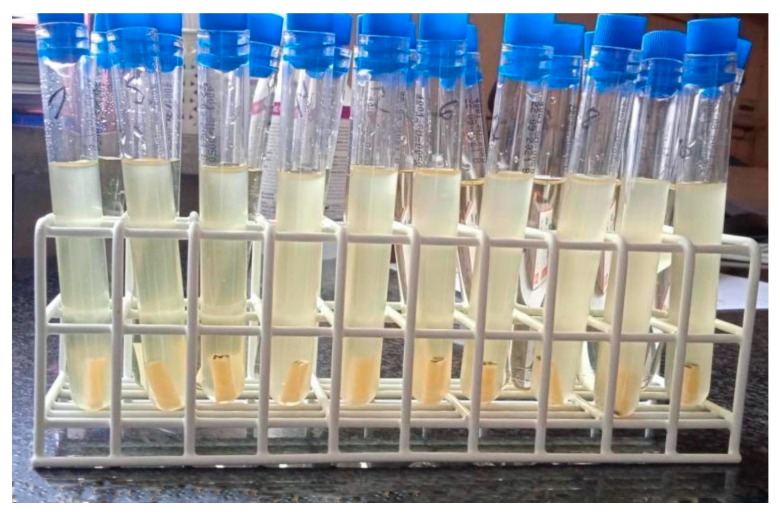
Analysis of biofilm formation using the *C. albicans* strain—qualitative method.

Using a modified Richards qualitative method [22], the production of bacterial and fungal biofilms on the surface of samples of zirconium oxide material was studied. Sterile samples of prosthetic materials were added to TSB (TrypticSoyBroth) medium (BD, Haidelberg, Germany) after a 24 h culture yielded a suspension with a density of 1.0 on the McFarland scale (Figure 3). The samples were incubated for 24 h at 37 °C. Following this time, they were rinsed three times with NaCl, and then 1 drop of 1% 2,3,5-triphenyltetrazolium chloride (TTC) solution was added. Once more, the samples were incubated at 37 °C for 24 h. Every test was run in triplicate. An intensity of color scale with three levels was used to interpret the data.

#### 2.3.2. Quantitative Method

**Figure 4:**
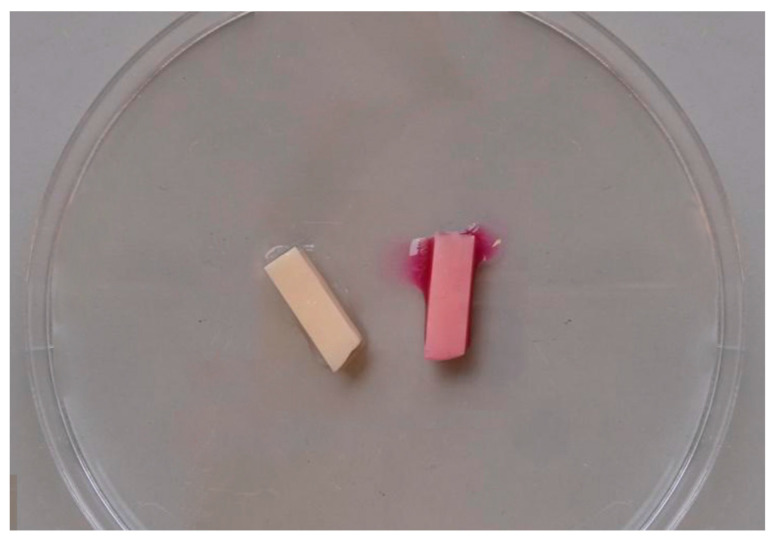
Analysis of biofilm formation using the *C. albicans* strain—quantitative method.

In accordance with Mączyńska et al. [23], the quantitative method (Figure 4) was utilized to investigate the development of bacterial and fungal biofilm on the surface of zirconium oxide material samples. Following the same procedure as in the qualitative approach, a suspension with a density of 1.0 on the McFarland scale was made. The examined materials were then added to the suspension and incubated for 24 h at 37 °C. Next, to separate the biofilm cells, the prosthesis samples were shaken for 60 s in a 0.5% saponin solution. In this manner, the suspension was prepared and then inoculated in 100 µL dilutions successively onto Columbia agar with 5% sheep blood (bioMérieux, Craponne, France) and Sabouraud’s medium (bioMérieux, Craponne, France). The incubation was then repeated for a full day at 37 °C. The measurement was performed by counting the number of colonies on the substrates on which individual dilutions were sown.

### 2.4. Statistical Analysis

The mean and standard deviation for a particular set of ceramics were computed for the descriptive analysis of the data acquired using the quantitative approach. The data distribution’s normality was evaluated using the Student’s *t*-test (*p* < 0.01). Data were dispersed in a typical manner. The average results of both materials were compared using the ISO 2854 standard [24].

## 3. Results

The qualitative and the quantitative method were used to measure the formation of biofilm on the surface of milled and 3D printed zirconia samples. The study findings indicate that variations exist in the evaluation of biofilm between the qualitative and quantitative approaches; nonetheless, in both approaches, the total absence of biofilm is not detected.

### 3.1. Qualitative Method

According to the hue of the tested samples, the following classification of results was used in the Richards method:

- (-)—absence of cells (non-biofilm forming strain);
- (+)—10^3^–10^4^ CFU/mL (strain with poor biofilm formation);
- (++)—10^5^–10^6^ CFU/mL (strain with strong biofilm formation);
- (+++)—10^7^–10^8^ CFU/mL (strain with very strong biofilm formation).

The results of the qualitative method are presented in Table 1.

The *C. albicans* strain was classified as very strongly biofilm-forming (+++) (Figure 1), but no significant differences were observed between 3D printed and milled zirconium oxide. The bacterial strains included in the investigation showed weaker biofilm formation, although there were no appreciable variations in biofilm formation on the surfaces of milled and 3D printed zirconium oxide.

### 3.2. Quantitative Method

The quantitative method’s assessment of biofilm formation results matched the qualitative method’s findings. There were minimal differences in the occurrence of biofilm on materials containing 3D printed (Table 2) and milled zirconium oxide (Table 3).

### 3.3. Statistical Analysis

**Figure 5:**
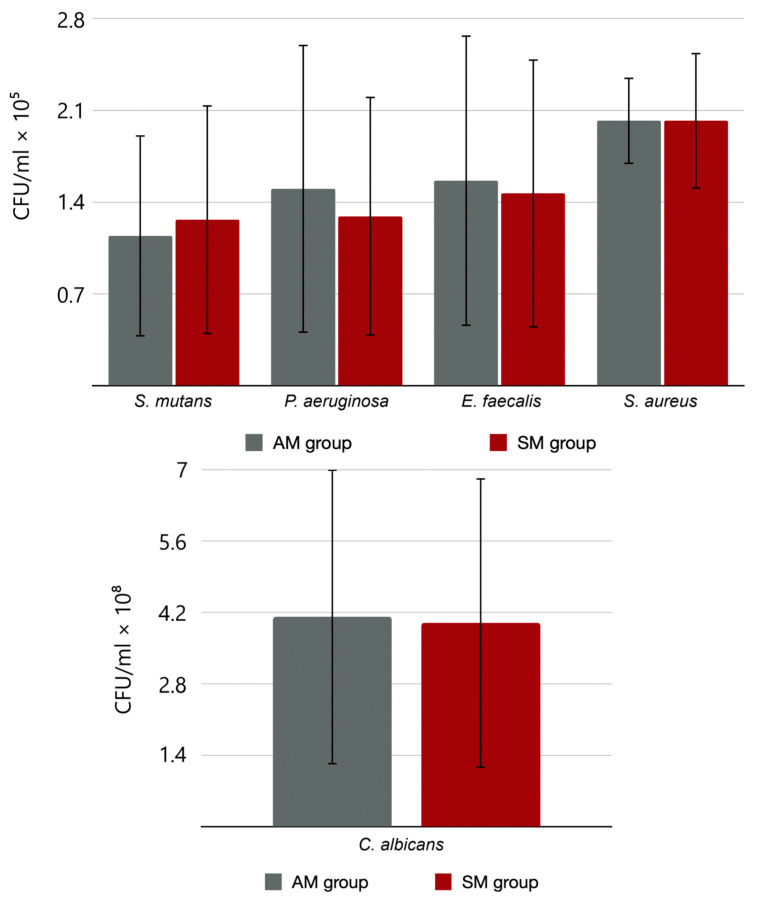
Results of means and standard deviations for bacterial and fungi strains obtained using the quantitative method.

The results of biofilm formation in the quantitative method for all strains in both materials show differences, but at the significance level of *p* < 0.01, they are not statistically significant. The mean CFU/mL (colony forming unit) and its standard deviation for individual microorganisms are shown in Figure 5.

## 4. Discussion

Currently, the material used in dentistry faces a number of challenges that it must meet. These include sufficient mechanical strength, high aesthetic requirements of the patient, as well as biocompatibility with oral tissues. Zirconium oxide, known for years as a material in dentistry, meets all these needs; therefore, its new substitute, zirconium oxide, made using 3D printing technology, must also meet these requirements. For this reason, the authors of the study conducted a qualitative and quantitative analysis of biofilm formation by the most common microorganisms found in the oral cavity.

It has long been known by microbiologists and dentists that *Candida albicans* can be found in the oral cavity [25]. It normally occurs in a healthy mouth and does not cause any adverse reactions. Unfortunately, when its balance is disturbed and the number or virulence of the fungus increases, it may lead to infection and then to the development of oral candidiasis [26]. In our own study, both tested materials were not subjected to any surface treatment to see how microorganisms behave on the surface immediately obtained after the production process. Statistical analysis (*p* < 0.01) confirmed the null hypothesis based on the results of quantitative tests that the difference in the intensity of biofilm production by *Candida albicans* is not statistically significant. Research by Cepic et al. [27] showed that a significant increase in the adhesion of *Candida albicans* fungi can be observed on glazed surfaces compared to those that have been previously polished. They also indicate that the factor that may increase this adhesion is probably mucin found in saliva. The same surface dependence occurs not only with fungi, but also with biofilms created by bacteria [28]. Therefore, it can be concluded that in order to reduce the formation of biofilm, the zirconium oxide surface should always be polished beforehand [29]. In turn, Khattar et al. [30] noticed that zirconium oxide as an addition to another material in low concentration (0.5%) not only does not increase the roughness of the material, but also significantly reduces the adhesion of fungi to the surface of the material compared to samples from other groups of the author’s study, which additionally confirms the information about the biocompatibility of zirconium oxide.

Oral bacteria play a key role in maintaining oral health, but they can also contribute to a variety of health problems [31]. Some bacteria in the mouth can contribute to the formation of dental plaque, which in turn leads to unpleasant breath odor and increases the risk of tooth decay and gum disease. This may lead to inflammation, such as gingivitis, or a more advanced stage of the disease, i.e., periodontitis. Bacteria can also accumulate in the spaces between teeth and gums, causing periodontal infections, which can be painful and lead to tooth loss. Their presence on prosthetic restorations or their connection with tooth tissues may also lead to the development of various types of diseases, which is why it is so important to check how intensively biofilm is formed on the surface of the material from which dental work is made. For this reason, the authors also decided to check whether there are differences in the adhesion of individual bacteria between milled and 3D printed zirconium oxide. Our own research showed no differences between the intensity of biofilm formation by bacteria in the case of both materials. This is advantageous because the new material can compete in this respect with milled zirconium, which has been available on the market for years. As research shows when comparing the intensity of biofilm among titanium, PEEK (polyether ether ketone) and zirconium oxide, the latter among those tested obtained the lowest rate of adhesion of microorganisms to its surface [32]. This is also confirmed by research by other authors [33], taking into account the fact that in the case of structures such as dental implants, although the initially formed biofilm accumulates in smaller amounts on zirconium implants than, for example, their titanium counterparts, in the event of their aging, this difference is reduced. It is more difficult to eradicate bacteria from the prosthetic restoration when the surface area of each material in contact with the mouth cavity is increased. Studies on various materials used in the dental industry [16,17] have shown that with increasing roughness, the adhesion of bacteria occurring in the oral cavity also increases. It follows that the main role in limiting the formation of biofilm should be played by polishing the surface of the prosthetic restoration. For zirconium oxide made using 3D printing technology, this can be created by extending the printing layer and reducing the porosity of the final product in order to achieve the highest quality restoration [34].

Other factors may also influence the adhesion of microorganisms to surfaces, one of which is the method of sterilization of the material surface. Dry sterilized samples show a significantly lower amount of biofilm on the zirconium oxide surface, while irradiation with UVC or gamma rays, among others, results in a greater multiplication of microorganisms. Some authors [35] associate this phenomenon with the increased hydrophilicity of the material, which may result in faster bacterial reproduction in a more humid environment.

All the above-described features prove the high biocompatibility of zirconium oxide, which, after additional treatments, can be further increased, and the colonization of its surface by oral microorganisms can be slowed down. Additionally, a new material in the form of printed zirconium oxide gives us the opportunity to create complex designs, such as implants or specialized denture connectors. We can also save some of the material that is unfortunately lost during standard milling production. In the future, after additional research and the spread of zirconium oxide in 3D printing technology, it may become a widely used material, not only due to the speed of the production process, but also to the creation of not only solid, but also partially porous elements [36].

### Limitations of the Study

The aim of the study was to demonstrate differences between materials in terms of microbial adhesion. Three-dimensional printing is still a new technology in dentistry, especially zirconium oxide printing. This is a technology that is constantly developing, and new manufacturers are constantly appearing on the market, making elements using this technology, as well as using other materials from which prosthetic restorations can be made. One such material is lithium disilicate, which has recently become an increasingly used material in restorative dentistry, among others, by the company Lithoz (Wien, Austria) [37]. The authors plan further research comparing this type of material with zirconium oxide made using 3D printing technology.

Another challenge facing the introduction of a new material to the market is the need for it to obtain ISO 13485 approval [38], which would permit its application in the healthcare sector. There are over 700 other species of bacteria and fungi in the oral cavity; that is why it would be impossible to check all of them in the study. Therefore, only the most popular species were used; so, the authors cannot guarantee that with a different type of microorganism, there would not be greater differences between the results obtained from the comparison of both materials.

The study’s authors intend to investigate the development of biofilm on the surface of other additively manufactured materials in the future, such as lithium disilicate or zirconium oxide produced by other manufacturers than the material utilized in this study. This is caused by the 3D ceramic printing industry’s ongoing development, which raises hopes for advancing technology and, consequently, lowering the cost of producing these materials in the future.

## 5. Conclusions

The results of the intensity of biofilm formation on the surface of zirconium oxide made using additive and milling technology show minimal differences that are not statistically significant. This indicates the potential for a wider introduction of 3D printed zirconium oxide into clinical practice.

## Figures and Tables

**Table 1:** Biofilm formation on zirconium oxide surfaces—qualitative method.

Strains Used in Biofilm Analysis on 3D Printed Zirconium Oxide Samples				
*S. mutans*	*P. aeruginosa*	*E. faecalis*	*S. aureus*	*C. albicans*
+	+	+	+	+++
Strains Used in Biofilm Analysis on Milled Zirconium Oxide Samples				
*S. mutans*	*P. aeruginosa*	*E. faecalis*	*S. aureus*	*C. albicans*
+	+	+	+	++

**Table 2:** Analysis of the impact of 3D printed zirconium materials on *S. mutans*, *P. aeruginosa*, *E. faecalis*, *S. aureus*, and *C. albicans* strains—quantitative method.

Trial Number	*S. mutans*	*P. aeruginosa*	*E. faecalis*	*S. aureus*	*C. albicans*
	CFU/mL				
1	1.8 × 10^5^	2.1 × 10^5^	2.5 × 10^5^	1.6 × 10^5^	6.1 × 10^8^
2	1.3 × 10^5^	1.5 × 10^4^	2.5 × 10^5^	2.5 × 10^5^	6.8 × 10^8^
3	2.1 × 10^5^	2.1 × 10^5^	2.3 × 10^4^	1.9 × 10^5^	5.5 × 10^7^
4	2.6 × 10^4^	2.6 × 10^4^	1.8 × 10^4^	2.3 × 10^5^	6.5 × 10^8^
5	2.5 × 10^4^	2.9 × 10^5^	2.4 × 10^5^	1.8 × 10^5^	6.2 × 10^7^

**Table 3:** Analysis of the influence of milled zirconium materials on *S. mutans*, *P. aeruginosa*, *E. faecalis*, *S. aureus*, and *C. albicans* strains—quantitative method.

Trial Number	*S. mutans*	*P. aeruginosa*	*E. faecalis*	*S. aureus*	*C. albicans*
	CFU/mL				
1	2.2 × 10^5^	2.1 × 10^5^	2.3 × 10^5^	1.3 × 10^5^	6.7 × 10^8^
2	1.7 × 10^5^	1.4 × 10^4^	2.2 × 10^5^	1.8 × 10^5^	5.8 × 10^8^
3	2.0 × 10^5^	2.1 × 10^5^	2.1 × 10^4^	2.9 × 10^5^	5.4 × 10^7^
4	1.8 × 10^4^	2.2 × 10^4^	2.2 × 10^4^	2.1 × 10^5^	6.4 × 10^8^
5	2.5 × 10^4^	1.9 × 10^5^	2.4 × 10^5^	2.0 × 10^5^	5.2 × 10^7^

## Data Availability

All the raw data are available from corresponding author on a reasonable request.
